# Is Adiponectin Related to Orofacial Clefts?

**Published:** 2012-01-01

**Authors:** S Khazaei, Sh Kazemi, M Khazaei

**Affiliations:** 1 Dental Student, Student Research Committee, School of Dentistry, Isfahan University of Medical Sciences, Isfahan, Iran; 2 Fertility and Infertility Research Center, Kermanshah University of Medical Sciences, Kermanshah, Iran

**Keywords:** Cleft lip and palate, Diabetes mellitus, Adiponectin

Dear Editor,

Orofacial clefts are the most common congenital anomalies of the head and neck and its incidence ranges from 1/500 to 1/2000 live births, depending on populations.[1] Etiology of these anomalies is multifactorial and includes both environmental and genetic factors. Many teratogenic agents and factors in pregnancy are claimed to cause clefting, such as maternal smoking and hypoxia[2] and diabetes mellitus.[3][4] The pivotal role of diabetes mellitus and maternal obesity on the incidence of orofacial clefts has been discussed previously.[5]

Adiponectin is 244-amino acid collagen-like polypeptide that is secreted by adipocytes and acts as an anti-inflammatory hormone and insulin sensitizer.[6] Adiponectin exists in at least two forms, low molecular weight oligomer that is hexamers (two trimers) and high molecular weight oligomer consisting of four to six tirmers.[7] Plasma concentration of this polypeptide in human blood ranges from 3 to 30 μg/ml and accounts for 0.05% of total plasma protein.[8]

Findings from animal studies and metabolic studies in human suggests that adiponectin has different properties, such as suppression of hepatic gluconeogenesis, stimulation of fatty acid oxidation in the liver, glucose uptake in skeletal muscle and stimulation of insulin secretion.[8][9] According to most of the studies in different populations, higher adiponectin level is associated with a lower risk of diabetes[10] and low plasma level of adiponectin is associated with increased insulin resistance both in children and adults.[11]

As we know, diabetes mellitus is one of the major risk factors of orofacial clefts.[3] So it is suggested that lower adiponectin levels in diabetic pregnant women takes part as a risk factor for orofacial clefts. This hypothesis could be assessed by screening pregnant women with diabetes mellitus for serum adiponectin level, and then investigating the incidence of cleft lip and/or palate in their children in comparison to control group.

According to our knowledge, diabetes mellitus is one of the important risk factors of orofacial clefts.[3] Adiponectin is secreted by adipocytes and increases insulin sensitivity. Higher levels of adiponectin are associated with lower risk of diabetes mellitus and the dose-response relation is consistent. If the association between adiponectin level and orofacial clefts in pregnant mothers can be verified, then one of the major risk factors of orofacial clefts could be achieved. These evidences suggest adiponectin level can be used as a screening marker for early diagnosis of obesity related abnormalities.
